# The Influence of Thermal Treatment of Activated Carbon on Its Electrochemical, Corrosion, and Adsorption Characteristics

**DOI:** 10.3390/molecules29204930

**Published:** 2024-10-18

**Authors:** Andrzej Świątkowski, Elżbieta Kuśmierek, Krzysztof Kuśmierek, Stanisław Błażewicz

**Affiliations:** 1 Institute of Chemistry, Military University of Technology, ul. Gen. S. Kaliskiego 2, 00-908 Warsaw, Poland; 2 Faculty of Chemistry, Institute of General and Ecological Chemistry, Lodz University of Technology, ul. Zeromskiego 116, 90-924 Lodz, Poland; 3 Department of Biomaterials and Composites, AGH University of Science and Technology, al. Mickiewicza 30, 30-059 Cracow, Poland

**Keywords:** heat-treated activated carbon, 2,4-D adsorption, morphology, electrochemical characteristics, corrosion

## Abstract

Activated carbons can be applied in various areas of our daily life depending on their properties. This study was conducted to investigate the effect of thermal treatment of activated carbon on its properties, considering its future use. The characteristics of activated carbon heat-treated at temperatures of 1500, 1800, and 2100 °C based on its future use are presented. The significant effect of the treatment temperature on morphological, adsorption, electrochemical, and corrosion properties was proved. Increasing the temperature above 1800 °C resulted in a significant decrease in the specific surface area (from 969 to 8 m^2^·g^−1^) and material porosity—the formation of mesopores (20–100 nm diameter) was observed. Simultaneously, adsorption capability, double layer capacity, and electrochemically active surface area also decreased, which helped to explain the shape of cyclic voltammograms recorded in 2,4-dichlorophenoxyacetic acid and in supporting electrolytes. However, a significant increase in corrosion resistance was found for the carbon material treated at a temperature of 2100 °C (corrosion current decreased by 23 times). Comparison of morphological, adsorption, corrosion, and electrochemical characteristics of the tested activated carbon, its applicability as an electrode material in electrical energy storage devices, and materials for adsorptive removal of organic compounds from wastewater or as a sensor in electrochemical determination of organic compounds was discussed.

## 1. Introduction

Carbon materials are present in almost all aspects of our daily lives. Activated carbons (ACs) are defined as amorphous carbonaceous materials that are characterized by high porosity and high specific surface areas, with values typically between 500 and 3900 m^2^·g^−1^, and low content of minerals but high content of carbon [1,2]. The surface area of carbon materials can be significantly increased by their activation with physical and chemical methods [3,4]. Physical methods include heat treatment at the specified temperature followed by activation under a gas stream, also at the specified temperature, while chemical methods include impregnation of a carbon material in chemical-activating agents followed by carbonization under an inert atmosphere [5].

ACs are usually classified into four groups due to their physical forms: powder ACs, granular and extruded ACs, fibrous ACs, and cloth ACs [6,7]. ACs’ versatile applications are due to their unique diversity in properties depending on the method of their synthetization and activation. Commonly, ACs are made from coal or charcoal. In the last few years, renewable resources have garnered great interest as potential raw precursors for AC production. Investigations have focused on agricultural wastes and lignocellulosic biomass residues, including rice, coconut, hemp, corn, caraway seed, palms, etc. [5,8,9,10,11,12,13,14]. The proper choice of precursor material plays a key role in the properties of ACs. Activated carbons are widely applied in water, air, and soil purification as adsorbents [15,16,17], supports for various catalysts [18,19], materials for hydrogen storage systems [20], and electrical energy storage devices [21].

Activated carbons can be applied as electrode materials not only in electrochemical capacitors (energy storage devices) but also in electrochemical systems for wastewater pollutant degradation. Thus, ACs can simultaneously play the roles of adsorbent and electrode [22,23]. Activated carbons used as adsorbents can also be electrochemically regenerated [24]. Regardless of the electrochemical process, the electrochemical and corrosion characteristics of ACs are important in achieving adequate results in wastewater treatment. The choice of raw precursor and its activation method certainly influence the electrochemical and adsorption properties and stability of the activated carbons.

Most of the current research has focused on preparation and activation methods for carbon materials and their adsorption characteristics [25,26,27]. There have only been a few reports on the comparison of the adsorption properties of carbon materials with their physicochemical [28,29], structural [30,31], or electrochemical characteristics [30,32,33]. Nevertheless, the comparison of adsorption, electrochemical, and stability or corrosion characteristics of activated carbon materials has not been reported. Such comparison should be useful for making an adequate choice of activated carbon for proper application.

Possible applications of activated carbon materials in water and wastewater treatment with various methods can define model compounds chosen for adsorption and electrochemical tests. 2,4-dichlorophenoxyacetic acid (2,4-D) is the most commonly used herbicide among plant protection products. 2,4-D is poorly biodegradable and is often found in aquatic environments as well as in wastewater [34]. It has been proved that this pesticide can not only pose a threat to aquatic life but can also be toxic to animals and humans. This compound is classified as carcinogenic and mutagenic and can have a negative effect on the central nervous system and kidneys. The development of an effective way of reducing the amount of this toxic contaminant in water reservoirs has become an urgent issue. Different methods of 2,4-D degradation by adsorption [25,35,36,37] and electrochemical methods [35,38,39] were tested; however, they still need further development.

The aim of this work was to investigate the effect of thermal treatment of activated carbon in an inert atmosphere on its properties. An increase in heat treatment temperature from 1500 to 2100 °C can significantly change the morphological, electrochemical, and corrosion characteristics of the tested materials. These changes in AC characteristics are particularly important for further application of ACs, e.g., as electrode materials in the degradation of organic compounds present in industrial wastewater, as sensors in the determination of organic and inorganic pollutants, or in energy storage devices.

## 2. Results and Discussion

### 2.1. Morphological Characterization of ACs

**Figure 1:**
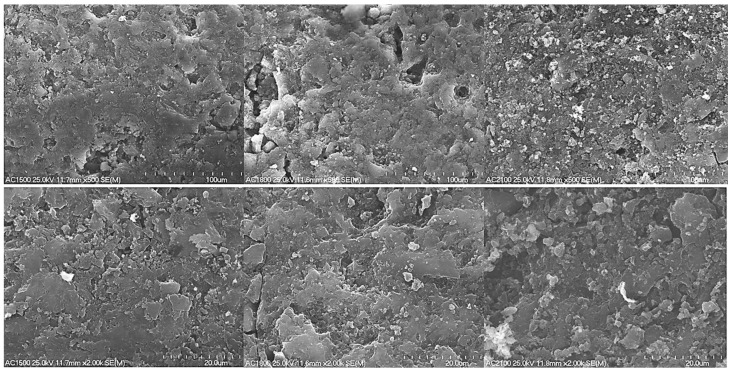
SEM images of the activated carbon samples (AC1500, AC1800 and AC2100) recorded at magnifications of 500 (**top**) and 2000 (**bottom**).

Changes on the surface of activated carbons were determined by recording SEM images under different magnifications. The effect of heat-treatment temperature on the surface of the activated carbon materials is presented in Figure 1.

An increase in the heat-treatment temperature from 1500 to 1800 °C resulted in no significant changes on the surface of the carbon materials (Figure 1). In the case of AC1500 and AC1800 samples, their surface was porous.

The results of thermogravimetric analysis of the tested activated carbons in an air atmosphere (the registered TG curves—Figure S1) reveal different temperatures at the beginning of carbon combustion: 630 and 656 °C for the AC1500 and AC1800 activated carbons, respectively [40]. In the case of TG curves registered in the CO_2_ atmosphere, the mass losses at a temperature of 850 °C decreased for AC1500 and AC1800 [41]. The above difference results from the different susceptibilities of the activated carbon surface to the Boudouard reaction. All these differences observed during thermogravimetric analyses result from the increasingly ordered structure of the carbons with the increasing heating temperature.

The FTIR spectra of all AC samples presented and described in the paper [42] prove changes in the chemical properties of their surfaces.

However, increasing the heat treatment temperature to 2100 °C caused the appearance of small aggregates with a microcrystalline structure. Results of XRD and Raman spectroscopy measurements performed for carbon materials heat-treated at temperatures of 1500, 1800, and 2100 °C, presented in the papers [42,43], proved that 1800 °C can be regarded as the transition point above which transformation of the amorphous particles to crystalline structures starts. The XRD spectra recorded for the AC samples [42,43] presented asymmetric diffraction bands in the range of 2*θ* from 20 to 30° and from 40 to 50°. The first diffraction band was ascribed to the (002) reflections in graphite crystallites. The second diffraction band (40–50°) was weaker and corresponded to the overlapping of (100) and (101) features. These features were found to be attributed to the possible presence of graphitic phase or small amounts of inorganic contaminations not removed during the demineralization of the sample and were weaker for AC2100. The analyses proved that the mean values of interlayer spacing (0.37 nm) did not change significantly with the increase in the heat-treatment temperature up to 1800 °C, causing small ordering and crystallinity of the carbon sample.

However, for the carbon sample heated at 2100 °C, a mean interlayer spacing value of 0.342 nm was obtained. Due to an increase in heat treatment temperature, the height of crystallite *Lc* increases from 1.1 nm at 1800 °C to 4.2 nm at 2100 °C.

The Raman spectra recorded for AC samples [42] revealed the presence of two broad bands at around 1350 and 1600 cm^−1^. The D band (1300 cm^−1^) was attributed to disorder due to the lack of long-range order in amorphous and quasi-crystalline forms of carbon materials. The G band (1600 cm^−1^) corresponded to the E_2g_ mode in the basal graphene layer. For D and G, the Raman band ratio I_D_/I_G_ for all AC samples was calculated. The I_D_/I_G_ ratio decreased as a function of the heat treatment temperature as follows: 1.83, 1.81, and 1.77 for 1500, 1800, and 2100 °C, respectively. At temperatures higher than 1800 °C, the structure of the activated carbon starts to be more ordered.

**Figure 2:**
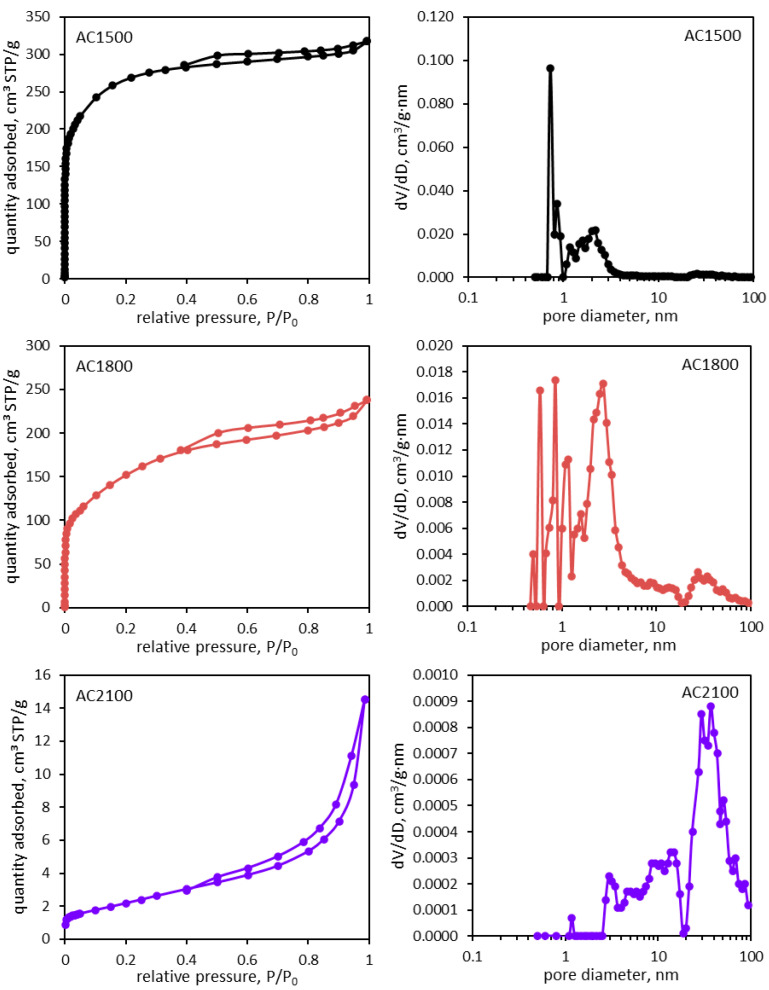
Nitrogen adsorption–desorption isotherms (**left side**) and pore size distribution dV/dD plots (**right side**) determined for the activated carbon samples.

Simultaneously, the porosity of AC2100 decreased significantly, which was proven by the measurement of adsorption–desorption isotherms of nitrogen at a temperature of 77.4 K (Figure 2). Parameters characterizing the porous structure of the activated carbon materials are presented in Table 1.

Results obtained based on nitrogen adsorption isotherms prove a significant decrease in the specific surface area of AC2100. Additionally, the volume of micropores and mesopores decreased significantly. However, in the case of AC1800, an observed increase of mesopore surface was clearly higher; this is visible in SEM images (Figure 1).

The distribution of pore volumes as a function of pore size determined for the tested activated carbon materials (Figure 2) reflects the above-described results.

In the case of AC1500 and AC1800, the pore volume is actually attributed to pores with a diameter not larger than 3 nm. Therefore, the porosity of these materials is mainly related to the presence of micropores. An increase of the heat-treatment temperature to 2100 °C results in quite a different distribution of pores and a significant decrease in their volume. Most of the pore’s volumes are attributed to their diameters being between 20 and 100 nm. These mesopores seem to be more accessible for the electrolyte and can participate in electrochemical oxidation or reduction as well as adsorption.

### 2.2. Adsorption Results

The results of adsorption experiments can determine the possible application of activated carbon; therefore, adsorption experiments were performed in a solution of 2,4-dichlorophenoxyacetic acid (2,4-D), which was used as a model organic compound often present in surface water and wastewater. Due to the adsorption properties of activated carbon, adsorption processes can be applied to the removal of organic compounds from the environment and to wastewater treatment.

**Figure 3:**
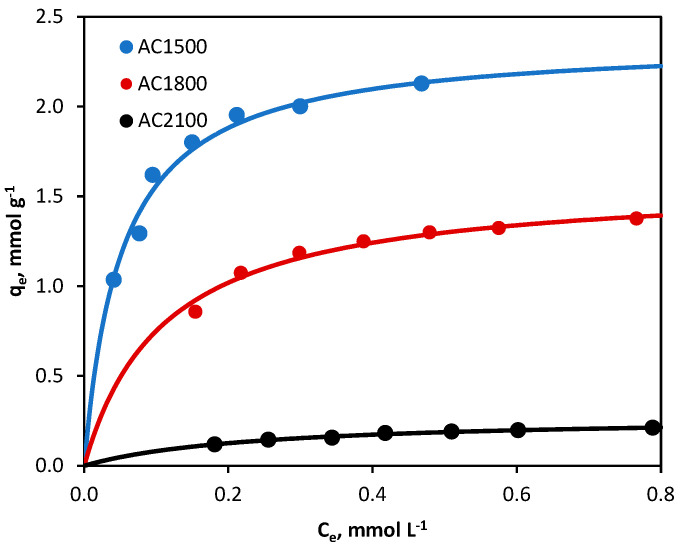
Adsorption isotherms of 2,4-D from 0.1 mol·L^−1^ KCl onto activated carbons (line: fitting of Langmuir model). Experimental conditions: 2,4-D initial concentrations = 0.3–1.0 mmol·L^−1^, activated carbon dosage = 0.5 g·L^−1^, temperature = 23 °C, pH = native (original).

The adsorption isotherms of 2,4-D from 0.1 mol·L^−1^ KCl on all three activated carbons are shown in Figure 3. KCl solution was chosen for the study taking into account the structure of 2,4-dichlorophenoxyacetic acid and the need for a supporting electrolyte application in electrochemical tests and processes.

The two most popular isotherm models, Freundlich and Langmuir, were used to describe adsorption. According to the Freundlich model, the adsorption is multilayer and the adsorbent surface is heterogeneous and non-uniform, while the Langmuir equation assumes adsorption in a monolayer on a homogeneous adsorbent surface. The linear forms of the Freundlich and Langmuir equations are given by Equations (1) and (2):

(1) $$\;\ln q_{e}={\ln K}_{F}+\frac{1}{n}{\ln C}_{e}$$

(2) $$\frac{C_{e}}{q_{e}}=\frac{1}{q_{m}}C_{e}+\frac{1}{q_{m}K_{L}}$$

where *C*_e_ is the equilibrium adsorbate concentration in the liquid phase (mmol·L^−1^), *q*_e_ is the adsorbate amount adsorbed at equilibrium (mmol·g^−1^), *q*_m_ is the maximum adsorption capacity (mmol·g^−1^), and *K*_L_ is the Langmuir constant (L·mmol^−1^), while *K*_F_ (mmol·g^−1^)(L·mmol^−1^)^1*/n*^ and *n* are the Freundlich constants.

The calculated parameters of the two isotherms together with the obtained correlation coefficients (*R*^2^), are shown in Table 2. By analyzing the *R*^2^ values obtained for the Freundlich and Langmuir models, it can be concluded that the adsorption of 2,4-D from the electrolyte solution on all three activated carbons tested is better described by the Langmuir equation (*R*^2^ ≥ 0.996). It can therefore be assumed that we are dealing with monolayer adsorption of the herbicide on a homogeneous adsorbent surface.

The obtained *q*_m_ (but also *K_L_*) values increase in the order AC2100 < AC1800 < AC1500, which is closely correlated with the specific surface area of the adsorbents tested (Table 1). Thus, the herbicide adsorbed best on AC1500 carbon, which has the highest BET surface area (969 m^2^·g^−1^), and least on AC2100 carbon, which has the lowest specific surface area (8 m^2^·g^−1^).

For an assessment of the adsorption properties of these carbons, it would be necessary to compare their adsorption capacities towards 2,4-D with other adsorbents described in the literature. Such a comparison can be found in the review paper [44]. However, in this case, such a comparison is difficult since the adsorption of the herbicide was not tested using water but a 0.1 mol·L^−1^ potassium chloride solution. The presence of an inorganic salt in a solution increases its ionic strength, and this in turn can affect adsorption by, among other things, altering the solubility of the adsorbate or the electrostatic interactions of the salt ions with both the adsorbate molecules and the adsorbent surface. The effect of inorganic salt concentration in solution (ionic strength of solution) is not obvious and depends on the physicochemical properties of both the adsorbate and the adsorbent and must be considered individually for each adsorbate/adsorbent system. For example, the ionic strength of the solution had no clear effect on the adsorption of 2,4-D and 4-chloro-2-methylphenoxyacetic acid (MCPA) on activated carbons prepared from rice straw [45] and on the adsorption of MCPA, 4-(4-chloro-2-methylphenoxy)butanoic acid (MCPB), and 2-(4-chloro-2-methylphenoxy)propanoic acid (MCPP) on activated carbons modified by HNO_3_ oxidation and heat treatment in ammonia at 900 °C [46]. On the other hand, it was observed that the adsorption of phenoxyacetic acid, 4-chlorophenoxyacetic acid, 2,4-D, and MCPA on lignite increases with the NaCl concentration in the solution [47]. A significant increase in the adsorption of 2,4-D in the presence of NaCl in the solution was also observed on two commercially available activated carbon types, Sorbo Norit and Ceca AC40 [48]. The authors attribute the increased herbicide adsorption in the presence of inorganic salt to a salting-out effect, which reduces 2,4-D solubility and increases adsorption on activated carbon. In the same paper [48], a slight reduction in the adsorption of MCPA in the presence of NaCl in solution was also observed, presumably due to a screening effect between the positively charged activated carbon surface and the negatively charged adsorbate molecules at the working pH.

The adsorption of 2,4-D from water on single-walled carbon nanotubes, reduced graphene oxide, and AC1800 was described in our previous work [49]. The adsorption capacities obtained for the Langmuir (*q*_m_) and Freundlich (*K*_F_) models were 1.652 mmol·g^−1^ and 1.462 (mmol·g^−1^)(L·mmol^−1^)^1/n^, respectively. They are therefore very close to the *q*_m_ and *K*_F_ values obtained in this study (1.588 mmol·g^−1^ and 1.564 (mmol·g^−1^)(L·mmol^−1^)^1/n^, respectively). Thus, by comparing the adsorption capacities of AC1800 from water [45] and from 0.1 mol·L^−1^ KCl presented in this paper, it can be assumed that the adsorption of 2,4-D on the tested activated carbons is not significantly affected by the presence of the inorganic salt in solution.

### 2.3. Corrosion Characterization of ACs

**Figure 4:**
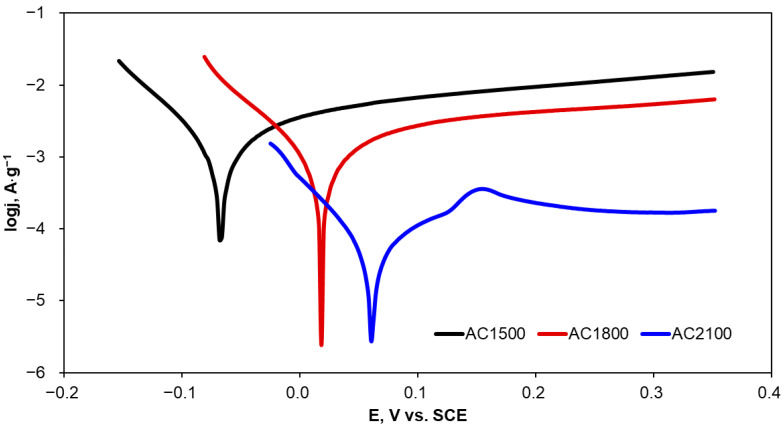
Potentiodynamic polarization curves recorded for the activated carbon samples in KCl solution (0.1·mol L^−1^).

The corrosion resistance of the activated carbons is particularly important since these materials can potentially be applied in the degradation of organic compounds present in industrial wastewater with the application of chemical, physical, and electrochemical methods. Therefore, corrosion characterization of the activated carbon materials was determined in KCl solution, which is often applied as a supporting electrolyte and often present in industrial wastewater. Evaluation of corrosion resistance was performed using potentiodynamic polarization preceded by the measurement of an open circuit potential (OCP). The polarization curves were recorded in the potential range of OCP ± 300 mV, with a relatively slow scan rate of 2 mV·s^−1^. Exemplary polarization curves are presented in Figure 4. Given the comparable geometric surface area but different masses of the carbon material samples, the current values were normalized to the mass of the activated carbon applied in corrosion experiments.

The corrosion parameters, i.e., OCP, corrosion potential (E_corr_), corrosion current density (j_corr_), polarization resistance (R_p_), and anodic and cathodic Tafel slopes (b_a_ and b_c_), determined from the polarization curves are listed in Table 3.

An increase in heat-treatment temperature of the tested carbon materials resulted in a clear increase not only in OCP but also in E_corr_. Given that E_corr_ is attributed to the susceptibility of a material to corrosion phenomena, it can be concluded that AC1500 corrodes more easily than AC2100. The R_p_ value shows material resistance to corrosion under specified conditions. AC2100 has a significantly higher (more than 20 times) R_p_ value in comparison with AC1500, which implies very high stability of the material. Moreover, this is reflected by the very low corrosion current density observed for AC2100; the lower the corrosion current density, the lower the corrosion rate observed for the specified material. Differences in corrosion resistance between AC1500 and AC1800 are clear but not so evident as in the comparison of these materials with AC2100. Increasing the heat-treatment temperature above 1500 °C resulted in a clear increase in corrosion resistance and a significant decrease in corrosion rate.

A comparison of Tafel slopes (b_a_ and b_c_) indicates that anodic and cathodic reactions during corrosion probably follow the same mechanism of anodic and cathodic reactions during corrosion regardless of the heat-treatment temperature of the carbon material.

**Figure 5:**
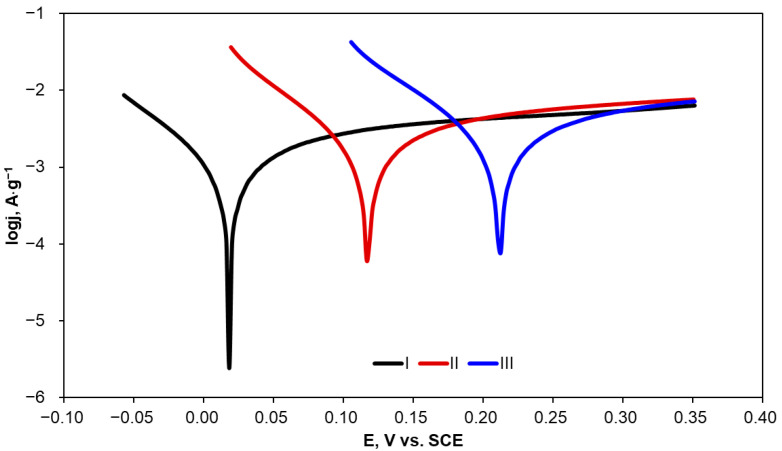
Exemplary potentiodynamic polarization curves recorded for AC1800 material in 0.1 mol·L^−1^ KCl in three consecutive measurements.

In order to compare the influence of the immersion time in KCl on the corrosion resistance of the tested carbon materials, three consecutive measurements were performed. Exemplary polarization curves are presented in Figure 5. The corrosion parameters determined for three consecutive measurements are compared in Table 4.

In the case of all tested carbon materials, a clear shift in OCP and E_corr_ towards more positive values was observed. This proves that corrosion of AC materials is more difficult. On the other hand, the corrosion current increases (1.5–2.3 times) in the case of all AC materials with increasing immersion time. This fact is reflected in the increasing values of R_p_. However, the j_corr_ value determined for the third measurement is still lowest for AC2100. Comparing E_corr_ and j_corr_ values proves that activation of carbon materials at 2100 °C results in the material with the highest resistance to corrosion phenomena in KCl solution, probably due to lowering of the amount of oxygen-containing functional groups, lowering porosity, and increasing crystallinity of the AC surface [42].

### 2.4. Electrochemical Characterization of ACs

**Figure 6:**
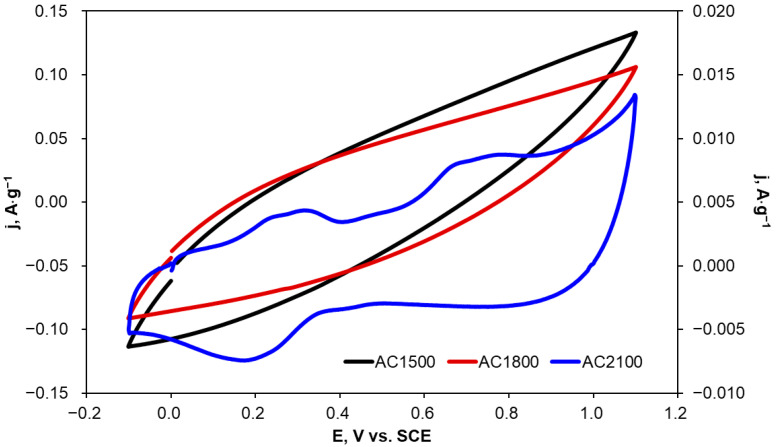
Cyclic voltammograms recorded on activated carbon samples in a 0.001 mol·L^−1^ 2,4-D solution (0.1 mol·L^−1^ KCl); v = 20 mV·s^−1^, AC1500 and AC1800 (left axis); AC2100 (right axis).

Carbon materials can potentially be used as electrode materials, e.g., for the determination of organic compounds or even for their electrochemical degradation. Therefore, voltammetric curves were recorded on carbon samples heat-treated at 1500, 1800, and 2100 °C in a 2,4-dichlorophenoxyacetic acid solution at a concentration of 0.001 mol·L^−1^ (0.1 mol·L^−1^ KCl). Example voltammograms are presented in Figure 6. The current density values presented in Figure 6 were normalized to the mass of the activated carbon samples applied as electrodes.

In the case of AC1500 and AC1800 electrodes, no peaks resulting from electro-oxidation or electro-reduction of 2,4-D were observed (Figure 6). Electro-oxidation currents observed at potentials higher than 0.4 V vs. SCE were slightly higher for AC1500 than for AC1800. This fact can be explained by differences in the surface areas of electrode materials, especially the outer one which is more accessible to the electrolyte solution. The lack of oxidation and reduction peaks is probably due to the high capacity of a double layer and simultaneous high contact resistance at the electrode/electrolyte interface resulting from the presence of oxygen functional groups [50].

AC2100 was heat-treated at a higher temperature (2100 °C) and was expected to reveal better electrochemical behavior towards 2,4-D oxidation due to a significantly lowering number of oxygen functional groups with increasing heat-treatment temperature. Cyclic voltammograms recorded for AC2100 show oxidation peaks in the potential ranges of 0.2–0.3 V and 0.7–0.8 V. These peaks can be attributed to 2,4-D electro-oxidation according to data in the literature [51,52,53].

In order to explain the behaviors of AC1500, AC-1800, and AC2100 in the 2,4-D solution, electrochemical characterization of these carbon materials was performed in KCl solution. This was followed by the determination of the number of electrochemically active sites and the porosity of the materials.

**Figure 7:**
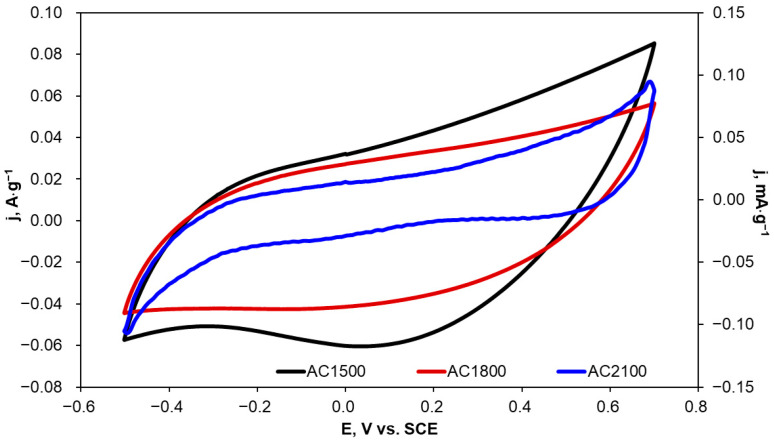
Cyclic voltammograms recorded in 0.1 mol·L^−1^ KCl for AC1500 (left axis), AC1800 (left axis), and AC2100 (right axis) carbon materials; v = 10 mV·s^−1^.

Due to the fact that KCl solutions are often applied as a supporting electrolyte in electrochemical oxidation and reduction processes, CV curves were recorded in 0.1 mol·L^−1^ KCl for the tested carbon materials with different scan rates and in the potential range from −0.5 to 0.7 V vs. SCE, i.e., between potentials of hydrogen and oxygen evolution. Example voltammetric curves recorded at a scan rate of 10 mV·s^−1^ are presented in Figure 7.

The current density values in the voltammograms presented in Figure 7 were normalized to the mass of carbon materials applied in the electrochemical measurements. These voltammograms were applied to the determination of a double layer capacity (*C*_dl_) at a potential of 0 V. Calculated results were also normalized to the mass of the activated carbon materials and are presented in Table 5 together with the specific surface area (*S_BET_*) determined for the tested materials. *C*_dl_ values were calculated according to the following Equation [54]:

(3) $$C_{\mathrm{dl}}=\frac{i_{C}}{v}$$

where *i*_C_ is the capacitive current and *v* is the potential scan rate.

An increase in heat-treatment temperature from 1500 to 1800 °C resulted in a decrease in C_dl_ by 25%. A similar decrease was observed for the S_BET_ parameter. However, a subsequent increase in the heat-treatment temperature to 2100 °C resulted in a significant decrease in C_dl_—by 1545 times; simultaneously, a clear decrease in S_BET_ was observed.

In the case of porous carbon materials, cyclic voltammograms recorded at different scan rates are expected to show a rectangular shape. The rectangular shape is characteristic of the pseudo-capacitive behavior of an electrode. Carbon materials heat-treated at 1500 and 1800 °C (AC1500 and AC1800) are characterized by cyclic voltammograms with a near-rectangular shape (Figure 7). This fact can be ascribed to high contact resistance at the electrode/electrolyte interface due to the existence of oxygen-containing functional groups [50]. However, a further increase in heat-treatment temperature to 2100 °C resulted in distorted voltammograms, which can be explained by the modification of the AC2100 surface by disappearing oxygen-containing functional groups and the formation of a more crystalline structure.

The voltammetric charge derived from the voltammograms recorded for the tested activated carbon samples in KCl solution (0.1 mol·L^−1^) can be attributed to the electroactive surface area (EASA) and corresponds to electrochemically active sites on the surface [55,56]. These sites can contribute to electrochemical reactions of oxidation and reduction. Cyclic voltammograms were recorded in the potential range between hydrogen and oxygen evolution potentials. The total charge q* can be determined by the integration of anodic and cathodic parts of cyclic voltammograms and calculated as the sum of anodic and cathodic charges. The total charge decreases with an increase in the scan rate and consists of “inner” and “outer” charges. The outer charge $q_{out}^{*}$ is ascribed to the external parts of the electrode surface, which are more accessible to reagents. At the same time, the inner charge $q_{in}^{*}$ is ascribed to internal parts of the electrode surface that are less accessible to reagents. In the range of low scan rates, $q_{in}^{*}$ is probably involved in a reaction with the supporting electrolyte. The charges $q_{tot}^{*}$ and $q_{out}^{*}$ can be determined with the application of the following Equations [57]:

(4) $$\frac{1}{q^{*}}=\frac{1}{q_{tot}^{*}}+A\sqrt{v}$$

(5) $$q^{*}=q_{out}^{*}+B\frac{1}{\sqrt{v}}$$

where q* is an integrated charge and A and B are constants. The inner charge can be calculated according to the following equation:

(6) $$q_{tot}^{*}=q_{out}^{*}+q_{in}^{*}$$

**Figure 8:**
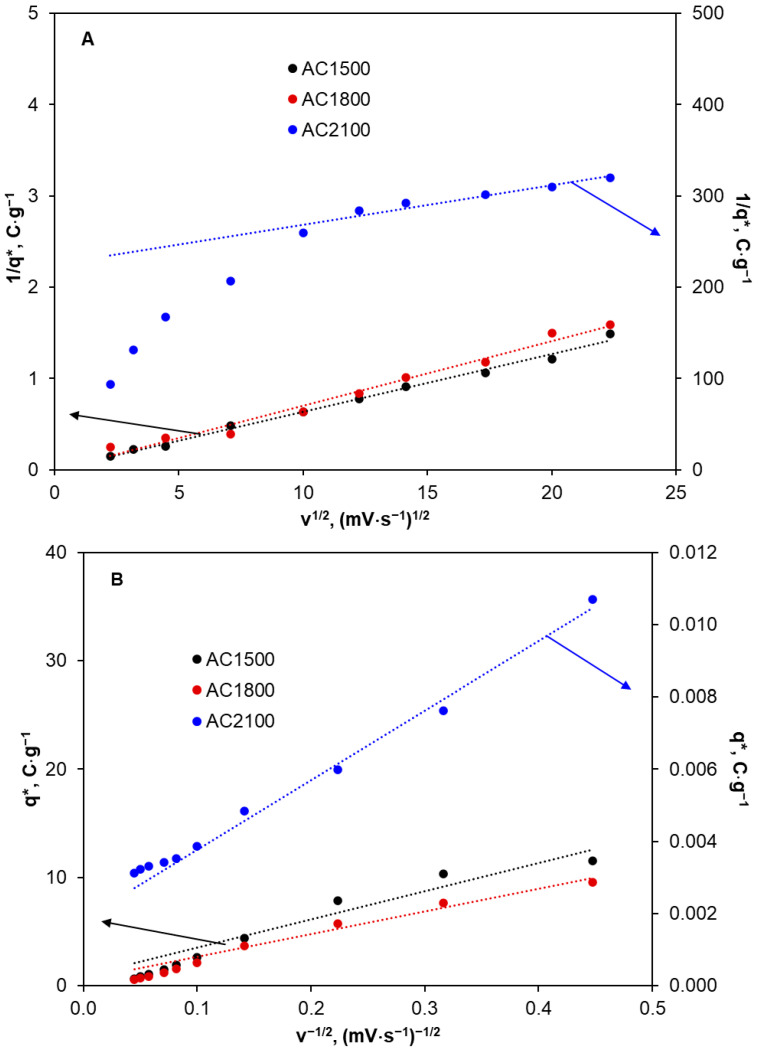
Dependences 1/q* vs. *v*^1/2^ (**A**) and q* vs. *v*^−1/2^ (**B**) determined for the activated carbons in KCl solution. AC1500 and AC1800—left axis, AC2100—right axis.

The dependencies 1/q* = *f(v*^0.5^) and q* = *f*(1/*v*^0.5^) determined for the activated carbon samples are presented in Figure 8 and the calculation results are shown in Table 6.

Given that voltammetric charge is related to the number of electrochemically active sites, it can be concluded that in the case of AC1500 and AC1800 (Table 6), most of the active sites (ca. 97–98%) are located inside pores and they are less accessible to electrolyte solutions. Only a small number of active sites are located on the external surface (2–3%) that is much more accessible. This is consistent with the results for S_BET_ and pore volume presented in Table 1. An increase in heat-treatment temperature to 2100 °C results in a significant decrease in the inner voltammetric charge and an increase in the outer voltammetric charge. In the case of AC2100, almost the same number of active sites are located in the outer and more accessible electrode surface (56%) as in the inner and less accessible surface (44%).

The porosity of electrode materials can be estimated from the value of the ratio $q_{in}^{*}$/$q_{tot}^{*}$ [58]. AC1500 and AC1800 show high porosity (ca. 97–98%). An increase in heat-treatment temperature to 2100 °C results in a decrease in porosity by more than 50%. However, the lower porosity of the activated carbon material and the higher number of electrochemically active sites on the outer surface in comparison with their number on the inner surface may explain the shape of the cyclic voltammograms recorded for the 2,4-D solution and presented in Figure 6. Lower oxygen-containing functional groups, lower double layer capacity, and a higher number of electrochemically active sites on the outer surface can result in the facilitation of 2,4-D electro-oxidation in the form of visible oxidation peaks in the cyclic voltammograms.

## 3. Materials and Methods

### 3.1. Materials and Chemicals

Three activated carbon samples labeled AC1500, AC1800, and AC2100 were applied in the investigations. Samples of granular (extruded) activated carbon were heat-treated at temperatures of 1500, 1800, and 2100 °C in an argon atmosphere. The heat-treatment activated carbon was demineralized with concentrated acids [27]. Commercial activated carbon (R3-ex) was obtained from Norit (Amersfoort, The Netherlands).

Pure 2,4-dichlorophenoxyacetic acid used for analysis was purchased from Sigma-Aldrich (St. Louis, MO, USA).

Potassium chloride (p.a.) was obtained from POCh (Gliwice, Poland). Stock standard solutions of 2,4-D were prepared in 0.1 mol·L^−1^ KCl.

A commercial extruded peat-based activated carbon Norit R3-ex was demineralized using concentrated HF and HCl acids. The portions of demineralized activated carbon were heated in a furnace chamber (Thermal Technology, HP, Minden, NV, USA) with special temperature programs. The samples were heated at a constant heating rate of 15 °C·min^−1^ in an argon atmosphere to different temperatures: 1500, 1800, and 2100 °C. After reaching the final temperature, they were kept at this temperature for 15 min and cooled at a rate of 20°·min^−1^. The activated carbon Norit R3-ex heat treatment programs are described in detail in the paper [43].

### 3.2. Material Characterization

The morphologies of the activated carbons were investigated by recording SEM images using a scanning electron microscope (S-4700, Hitachi, Tokyo, Japan). The sample surface was characterized by recording nitrogen adsorption–desorption isotherms measured at a temperature of 77.4 K using a Micrometrics ASAP 2010 volumetric adsorption analyzer (Norcross, GA, USA). Each measurement was preceded by degassing the sample under vacuum at 200 °C.

### 3.3. Batch Adsorption Experiments

The adsorption experiments were carried out using a batch method specified by the following procedure. A 0.15 g quantity of weighed potassium chloride and 10 mg of adsorbent were added to Erlenmeyer flasks containing 20 mL 2,4-D solution with an appropriate initial concentration (0.3 to 1.0 mmol·L^−1^). The final concentration of KCl in the solution was 0.1 mol·L^−1^. The mixtures prepared in this way were agitated at 23 °C at a constant speed of 100 rpm for 8 h. After this, the solutions were filtered through filter paper and the clear filtrate was analyzed for the herbicide residual in the solution. The concentration of 2,4-D in the solution was determined spectrophotometrically at the analytical wavelength λ = 283 nm (Varian Carry 3E series UV-Vis spectrophotometer, Palo Alto, CA, USA). The calibration curve for the determination of 2,4-D was linear (*R*^2^ = 0.9976) over the range of herbicide concentrations tested (0.05 to 1.0 mmol·L^−1^) and was described by the equation *y* = 1.628*x* + 0.0546. The amount of herbicide adsorbed at equilibrium (*q*_e_) was calculated from the following relationship:

(7) $$q_{e}=\frac{(C_{0}-C_{e})V}{m}$$

where *C*_0_ and *C*_e_ are the initial and equilibrium herbicide concentrations (mmol·L^−1^), respectively, *m* is the mass of the adsorbent sample (g), and *V* is the solution volume (L).

### 3.4. Electrochemical Measurements

Electrochemical measurements applied to carbon material characterization were performed in the three-electrode cell connected to the electrochemical workstation μAUTOLAB III (Metrohm Autolab B.V., Utrecht, The Netherlands). NOVA software ver. 2.1.6 was used for the analysis of recorded voltammetric curves and the determination of corrosion parameters. Carbon material samples in the form of 3 rods that were 5 mm high and had 2 mm diameters were attached to a platinum wire placed in a glass capillary. These electrodes with a geometric area of 1.17 ± 0.12 cm^2^ were used as the working electrode. A saturated calomel electrode (SCE) and platinum electrode were used as the reference and counter electrode, respectively. Before measurement, all liquid samples were deoxygenated with argon. During electroanalytical measurements, an argon blanket was kept over the solution’s surface to prevent diffusion of air inside the bulk solution.

The corrosion resistance of the carbon materials was determined in the supporting electrolyte KCl (0.1 mol·L^−1^) and was estimated using the electrochemical technique. After the immersion of a tested electrode in the supporting electrolyte, the measurement of open circuit potential (OCP) was conducted for 1 h or less if the OCP value was constant, i.e., the condition dE/dt ≤ 1 μV·s^−1^ was fulfilled. Next, the carbon electrodes were cathodically and anodically polarized in the potential range of OCP ± 200 mV with a scan rate of 2 mV·s^−1^.

The electrochemical behavior of 2,4-dichlorophenoxyacetic acid (2,4-D) on the tested carbon materials heated at different temperatures was determined using cyclic voltammetry (CV). Cyclic voltammograms were recorded at ambient temperature in the 2,4-D solution at a concentration of 1·10^−3^ mol·L^−1^ (0.1 mol·L^−1^ KCl). Potassium chloride was used as a supporting electrolyte.

Determination of the electroactive surface area (EASA) was performed in the KCI solution at a concentration of 0.1 mol·L^−1^ by recording voltammograms at scan rates in the range from 5 to 500 mV·s^−1^

## 4. Conclusions

The tested activated carbon materials showed a noteworthy influence of heat-treatment temperature on their morphological, adsorption, electrochemical, and corrosion characteristics, which is important for the choice of carbon material depending on its intended use.

The adsorption characteristics of ACs proved that AC1500 appeared to be the best adsorbent for 2,4-D. The simultaneous presence of inorganic salts should not significantly affect the adsorption of 2,4-D on all ACs.

The corrosion characteristics of ACs resulted in a conclusion that the increase in the heat-treatment temperature caused an increase in the corrosion resistance of the carbon material. The increase in immersion time in the electrolyte solution caused an increase in corrosion rate in the case of all tested activated carbon samples; however, AC2100 corroded much more slowly than the other materials.

The increase in temperature resulted in a decrease in the total volume of pores as well as in the volume of micropores, while the volume of mesopores increased with the increase in the temperature to 1800 °C and decreased at higher temperatures (2100 °C). Simultaneously, the pore distribution changed, and the formation of mesopores with diameters between 20 and 100 nm was observed. This can explain the shape of the cyclic voltammograms recorded in the 2,4-D solution.

Moreover, the double-layer capacity was significantly lower for AC2100 in comparison with AC1500 and AC1800. This can explain the lack of oxidation or reduction peaks in the cyclic voltammograms recorded at AC1500 and AC1800 in the 2,4-D solution and the pseudo-capacitive shape of voltammograms recorded in KCl. Determination of the electroactive surface area proved a significant decrease in the number of electrochemically active sites on the surface of AC2100 and a clear decrease in the porosity of this material. The increase in outer active sites in comparison with inner active sites located inside pores may explain the shape of the cyclic voltammograms recorded for the 2,4-D solution and the appearance of peaks attributed to the oxidation of this compound.

Comparing morphological, adsorption, corrosion, and electrochemical characteristics of the tested ACs, it can be concluded that AC1500 and AC1800 materials can be used as electrode materials in electrical energy storage devices and for removal of organic compounds from wastewater by adsorption, while AC2100 material should be applied as a sensor in the electrochemical determination of organic compounds.

## Figures and Tables

**Table 1:** Parameters of the porous structure determined for the activated carbon samples on the basis of nitrogen adsorption isotherms (S_BET_—specific surface area, V_mi_—micropore volume, V_me_—mesopore volume, V_tot_—total pore volume).

Carbon Material	S_BET_, m^2^·g^−1^	V_mi_, cm^3^·g^−1^	V_me_, cm^3^·g^−1^	V_tot_, cm^3^·g^−1^
AC1500	969	0.416	0.070	0.486
AC1800	551	0.235	0.125	0.360
AC2100	8	0.00034	0.022	0.0223

**Table 2:** The Freundlich and Langmuir model constants for adsorption of 2,4-D from 0.1 mol·L^−1^ KCl onto activated carbon.

Isotherm Model	Adsorbent		
	AC1500	AC1800	AC2100
Freundlich			
*K*_F_ ((mmol·g^−1^)(L·mmol^−1^)^1/n^)	2.922	1.564	1.061
1/*n*	0.298	0.292	0.275
*R* ^2^	0.910	0.921	0.966
Langmuir			
*q*_m_ (mmol·g^−1^)	2.370	1.588	0.277
*K*_L_ (L·mmol^−1^)	19.26	8.970	4.171
*R* ^2^	0.997	0.997	0.996

**Table 3:** Corrosion parameters of the activated carbon samples determined in 0.1 mol·L^−1^ KCl.

Carbon Material	OCP, V	E_corr_, V	j_corr_, A·g^−1^	R_p_, Ω	b_a_, V·dec^−1^	b_c_, V·dec^−1^
AC1500	0.133	−0.067	9.48·10^−4^	277	0.100	0.062
AC1800	0.217	0.018	5.05·10^−4^	366	0.077	0.051
AC2100	0.273	0.061	4.04·10^−5^	6153	0.085	0.053

**Table 4:** Corrosion parameters calculated for the activated carbons in 0.1 mol·L^−1^ KCl determined in three consecutive measurements.

Measurement	OCP, V	E_corr_, V	j_corr,_ A·g^−1^	R_p_, Ω	b_a_, V·dec^−1^	b_c_, V·dec^−1^
AC1500						
I	0.133	−0.067	9.48·10^−4^	277	0.100	0.062
II	0.210	0.010	1.23·10^−3^	193	0.099	0.053
III	0.298	0.101	1.47·10^−3^	152	0.099	0.050
AC1800						
I	0.217	0.018	5.05·10^−4^	366	0.077	0.051
II	0.317	0.117	8.82·10^−4^	222	0.082	0.054
III	0.403	0.212	1.17·10^−3^	193	0.091	0.064
AC2100						
I	0.273	0.061	4.04·10^−5^	6153	0.085	0.053
II	0.356	0.114	5.26·10^−5^	3139	0.046	0.045
III	0.380	0.128	8.55·10^−5^	2118	0.054	0.042

**Table 5:** The capacity of a double layer (*C*_dl_) compared with the specific surface area (S_BET_) determined for the activated carbon samples.

Carbon Material	*C*_dl_, F·g^−1^	S_BET_, m^2^·g^−1^
AC1500	4.5	969
AC1800	3.4	551
AC2100	2.2·10^−3^	8

**Table 6:** The values of voltammetric charges ($q_{tot}^{*}$, $q_{out}^{*}$, and $q_{in}^{*}$) calculated for the activated carbons from cyclic voltammograms recorded in KCl solution at various scan rates.

Carbon Material	$q_{tot}^{*}$, C·g^−1^	$q_{out}^{*}$, C·g^−1^	$q_{in}^{*}$, C·g^−1^	$q_{out}^{*}$ $/q_{tot}^{*}$	$q_{in}^{*}$ $/q_{tot}^{*}$
AC1500	129.37	2.10	127.27	0.02	0.98
AC1800	45.18	1.35	43.83	0.03	0.97
AC2100	4.60·10^−3^	2.56·10^−3^	2.04·10^−3^	0.56	0.44

## Data Availability

Data are contained within the article.

## Supplementary Materials

The following supporting information can be downloaded at: https://www.mdpi.com/article/10.3390/molecules29204930/s1, Figure S1: Thermogravimetric measurements of the activated carbons: unheated AC (blue line), AC1500 (red line), and AC1800 (black line).
