# Supplementary material for: The Influence of Thermal Treatment of Activated Carbon on Its Electrochemical, Corrosion, and Adsorption Characteristics
Source: Molecules. 2024 Oct 18;29(20):4930. doi: 10.3390/molecules29204930 (PMC11510475; doi:10.3390/molecules29204930)
Supplement: Supplementary file 1 [file molecules-29-04930-s001.zip › molecules-3195305-supplementary.pdf]

The Influence of Thermal Treatment of Activated Carbon on Its Electrochemical, Corrosion and Adsorption Characteristics

Andrzej Świątkowski [1], Elżbieta Kuśmierek [2,*], Krzysztof Kuśmierek [1] and Stanisław Błażewicz [3]

Due to the fact that the reference [41] is not available in electronic version, we present a figure with TGA curves as a supplement to TGA results presented in the paper.

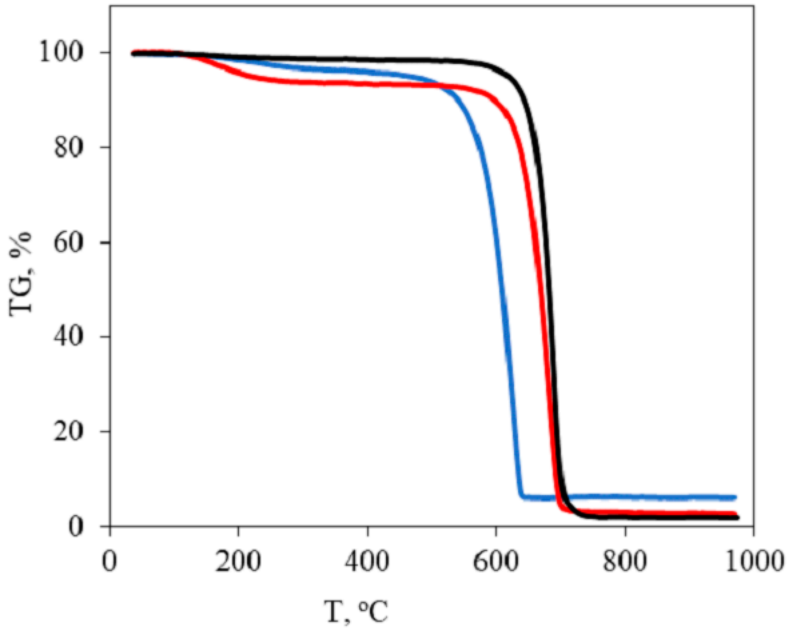

Figure S1. Thermogravimetric measurements of the activated carbons: unheated AC (blue line), AC1500 (red line) and AC1800 (black line).
